# Honoring Daniel Aberdam’s scientific and human legacy

**DOI:** 10.1038/s41419-025-07852-1

**Published:** 2025-07-24

**Authors:** Cédric Blanpain, Eleonora Candi, Alberto Gandarillas, Gerry Melino, Mauro Piacentini, Ruby Shalom-Feuerstein

**Affiliations:** 1 Laboratory of Stem Cells and Cancer, Université Libre de Bruxelles (ULB), Brussels, Belgium; 2 WEL Research Institute, Université Libre de Bruxelles (ULB), Brussels, Belgium; 3 Department of Experimental Medicine, TOR, University of Rome Tor Vergata, Rome, Italy; 4 Cell cycle, Stem Cell Fate and Cancer Laboratory, Institute for Research Marqués de Valdecilla (IDIVAL), Santander, Spain; 5 Institut national de la santé et de la recherche médicale, (INSERM), Délégation Occitanie, Montpellier, France; 6 Department of Genetics & Developmental Biology, The Rappaport Faculty of Medicine & Research Institute, Technion Integrated Cancer Center, Technion - Israel Institute of Technology, Haifa, Israel

**Keywords:** Embryonic stem cells, Skin stem cells, Cell lineage


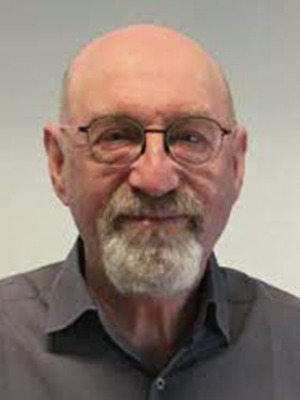

*And if the earthly no longer knows your name*,

*whisper to the silent earth: I’m flowing*.

*To the flashing water say: I am*.

(Rainer Maria Rilke, The Sonnets to Orpheus, II #28)

## In Memoriam: Daniel Aberdam (1954–2025)

Daniel Aberdam, a distinguished scientist and cherished friend, passed away on April 7th 2025. Daniel Aberdam was not only a brilliant researcher but also an inspiration to his generation—admired equally for his scientific achievements and for his vibrant personality, zest for life, and boundless enthusiasm.

After earning a B.A. in Biology from Pierre & Marie Curie University (Paris VI), he completed his M.Sc. and Ph.D. at the Weizmann Institute of Science in Rehovot under the mentorship of Leo Sachs, where he investigated the oncogenic potential of homeobox genes in leukemia. He then pursued postdoctoral research with Jean-Paul Ortonne in Nice, eventually becoming Director of Research (DR2) at INSERM U364 and U898. In 2006, he also established a productive French-Israeli binational laboratory at the Technion in Haifa, and in later years returned to Paris.

Daniel authored numerous landmark publications that have had a lasting impact on both scientific and clinical communities. Early in his career, he demonstrated that point mutations in the genes encoding Laminin 5 and integrin β4 cause junctional epidermolysis bullosa, a congenital skin blistering disorder. Among his many contributions, his pioneering studies on modeling skin and corneal lineage commitment using pluripotent stem cells stand out. His research evolved to focus on skin physiopathology, epidermal gene regulation, and stem cell biology. His group developed innovative cellular models derived from pluripotent stem cells that recapitulate both normal and pathological development of the skin and cornea. These models enabled the identification of key genes and signaling pathways, with particular emphasis on BMP4 and the transcription factor p63, which plays a central role in ectodermal dysplasia syndromes. His team was among the first to reconstruct skin tissue from pluripotent stem cells.

In recent years, Daniel turned his attention to therapeutic innovation. He investigated several small-molecule compounds, including PRIMA-1MET (APR-246), which led to clinical trials for patients with ectodermal dysplasia caused by mutations in p63. His work on the cornea also led to the identification of promising compounds for treating Pax6-related limbal stem cell deficiency, currently under investigation in preclinical models.

Daniel’s expertise and lasting contributions to the field were also reflected in his long-standing dedication as an editorial board member of several leading scientific journals, including Cell Death & Differentiation (since 2010), Stem Cells (since 2009), and Stem Cells & Cloning (since 2008).

Daniel was truly one of a kind. His passion, generosity, and humanity left a profound mark on all who knew him. He was a beloved friend, an inspiring mentor, and a vibrant presence in the scientific community. His enthusiasm was infectious, and his support unwavering.

## Alberto Gandarillas

I was fortunate to get to know Daniel because of our common interest in skin biology in France and because he was my mentor within the INSERM French biomedical Institute to which he belonged, when I obtained my position here. With sadness, I say goodbye to Daniel in his physical self, although his support and soul will remain with me, as with many other colleagues, no doubt. Although his duty as my INSERM tutor lasted only one year, he later always supported me and praised my work. He was always there and gave me advice whenever I asked. He helped me in many ways and was understanding when I had to make very difficult personal decisions. Scientifically, Daniel was always refreshing, inspiring and encouraging in our sometimes-hard endeavors. He was also a remarkable colleague in the skin field. I will miss Daniel in his physical self in many ways. He is probably the most supportive person I have encountered in my career. I can see his smile and friendly talk. Merci, Daniel, pour ton amitié et tout ce que tu nous as donné.

## Ruby Shalom-Feuerstein

Daniel was my postdoctoral mentor. He was very close to me—like a father or an older brother—and over the years, he became a close collaborator and a very dear friend.

Daniel, your generosity and deep curiosity for science made you a constant presence in my academic life. You followed my research closely for years, always offering thoughtful insights and sharing your vast experience with humility and warmth. Your support meant the world to me. You had a rare gift for bringing people together. You were the glue that connected scientists across disciplines and borders, creating new research hubs and fostering a sense of community wherever you went. With your absence, that unique sense of connection feels profoundly diminished.

Daniel, your spirit lives on in all of us—but your presence is sorely missed.

## Gerry Melino

Twice, Daniel, you came to Rome as a Visiting Professor with Edith, and those were truly special times—full of bonding, collaboration, and inspiration. We met with joint PhD students, launched provocative and innovative ideas, explored new restaurants and museums, and spent evenings together… ultimately becoming close friends. This is how the best science progresses—without boundaries. We also met several times in Haifa and Tel Aviv, and together we visited Aaron Ciechanover at his home. Ci manchi tantissimo!!!!

## Cédric Blanpain

Thank you, Daniel, for your enthusiasm, your kindness, and your support. We miss you, and we will always remember you as a very generous scientist who made important impacts in our field.

## Eleonora Candi

I was lucky to know you, Daniel, both as a collaborator and as a friend. I remember your suggestions and encouragement when we were applying for a grant. You were also a generous scientist, willingly sharing important reagents with me. Thank you, Daniel. I will deeply miss you as a colleague and friend.

We will miss him sorely. Our thoughts and deepest sympathy are with his close, beloved family. Our hearts remain close to Edith, his childre,n and grandchildren.

*Build me up of memory*

*loving and angry, tender and honest*.

*Let my loss build me a heart of wisdom*,

*compassion for the world’s many losses*

*Each hour is mortal*

*and each hour is eternal*

*and each hour is our testament*.

*May I create worthy memories*

*all the days of my life*.

(Debra Cash)

